# Prognostic value of TIM-1 expression in human non-small-cell lung cancer

**DOI:** 10.1186/s12967-019-1931-2

**Published:** 2019-05-28

**Authors:** Xiao Zheng, Kai Xu, Lujun Chen, You Zhou, Jingting Jiang

**Affiliations:** 1 Department of Tumor Biological Treatment, The Third Affiliated Hospital of Soochow University, Changzhou, Jiangsu 213003 People’s Republic of China; 2 Jiangsu Engineering Research Center for Tumor Immunotherapy, Changzhou, Jiangsu 213003 People’s Republic of China; 3 Institute of Cell Therapy, Soochow University, Changzhou, Jiangsu 213003 People’s Republic of China

**Keywords:** TIM-1, Lung cancer, Immunohistochemistry, RNAi, Prognosis

## Abstract

**Background:**

T-cell immunoglobulin and mucin domain 1 (TIM-1) is an important co-stimulatory molecule which serves as a surface marker for T cell activation, especially for Th2 cells. Recently, many studies have also shown that TIM-1 can be abnormally expressed in human cancers and may have a potential role in promoting cancer progression.

**Methods:**

The immunohistochemistry was used to examine the TIM-1 expression in human non-small-cell lung carcinoma (NSCLC) tissues. The cellular studies were performed to investigate the role of TIM-1 in the regulation of biological functions of human lung cancer cell lines.

**Results:**

We found that the TIM-1 expression was increased in human NSCLC tissues compared with the adjacent normal tissues, and the OS rate of NSCLC patients with higher TIM-1 expression was significantly lower compared with the ones with lower TIM-1 expression. The COX model showed that higher TIM-1 expression in lung cancer tissues could be used as an independent prognostic predictor for the patients. Furthermore, we depleted TIM-1 in NSCLC cell lines A549 and SK-MES-1, and the cellular functional studies also revealed that depletion of TIM-1 could significantly inhibit the cell viability as well as the abilities of migration and invasion. In addition, our microarray data showed that certain signaling pathways were altered and enriched after depletion of TIM-1. We subsequently verified that PI3K/Akt signaling pathway was involved in the TIM-1-mediated regulation of cellular functions in NSCLC cells.

**Conclusion:**

Our findings supported the notion that TIM-1 could serve as a potential therapeutic target for NSCLC.

## Background

Lung cancer is the most commonly diagnosed cancer and the leading cause of tumor-related death worldwide [1–3]. Non-small-cell lung carcinoma (NSCLC), which accounts for about 85% of all lung cancer cases, is another type of epithelial lung cancer other than small-cell lung carcinoma (SCLC) [4]. Surgical resection has been suggested as the conventional treatment for the patients with early-stage NSCLC [5]. For advanced NSCLC, although surgical therapy, chemotherapy, radiotherapy, targeted therapy and even combined therapy have been used, the 5-year overall survival (OS) still remains less than 15–20% due to the local recurrence or distant metastasis [6–8]. Recently, the immune checkpoint blockade therapy (ICBT), such as anti-programmed cell death 1 (anti-PD-1) therapy, anti-cytotoxic T-lymphocyte antigen 4 (anti-CTLA-4) therapy and anti-programmed cell death-ligand 1 (anti-PD-L1) therapy, have been shown to make a great breakthrough in the therapeutic strategies against human NSCLC [9–11]. Therefore, it’s of great interest for us to establish novel ICBT strategies and investigate the potential value of clinical application targeting some other immune checkpoint molecules, such as TIM-3 and LAG-3 [12, 13].

T-cell immunoglobulin and mucin domain 1 (TIM-1), also known as hepatitis A virus cellular receptor 1 (HAVcR-1) or kidney injury molecule 1 (KIM-1), is an important susceptibility gene for asthma and allergy, and it is preferentially expressed on Th2 cells and functions as a potent co-stimulatory molecule for T cell activation [14, 15]. As the first characterized member from TIM family, TIM-1 is initially found in monkeys and subsequently in humans as the HAVcR1 [16, 17]. Although lots of data have shown that TIM-1 has an essential role in the regulation of the T cell functions, however, it was also reported that TIM-1 play a critical role in the efficient clearance of apoptotic cells [18]. TIM-1 has been demonstrated to be expressed in numerous human tumors tissues [15]. In human Langerhans cell sarcoma (LCS), TIM-1 could be found in cancer cells, CK-18-positive epithelial cells and CD68-positive macrophages [19]. Liu et al. [20] have also reported that increased TIM-1 expression is found in human gastric cancer tissues compared with the normal gastric tissues at both the mRNA and protein levels, and high expression of TIM-1 can serve as a novel prognostic factor for gastric cancer. Moreover, urinary TIM-1 can also be found in patients with clear renal cell carcinoma, and its expression is significantly correlated with tumor characteristics [21].

Herein, in our present study, we aimed to examine the TIM-1 expression in both human NSCLC tissues and adjacent normal lung tissues, and further investigate the prognostic value and clinical implications of TIM-1 expression in NSCLC. Moreover, cellular studies were also performed to reveal the essential role of TIM-1 in functional regulation of human NSCLC cancer cells.

## Materials and methods

### Tissue samples

The NSCLC tissue arrays including squamous cell carcinoma (Catalog number: HLug-Squ150Sur-01) and adenocarcinoma (Catalog number: HLug-Ade180Sur-01) were purchased from Shanghai Outdo Biotech Co., Ltd. (Shanghai, P. R. China). In brief, 75 cases of squamous cell carcinoma and 90 cases of adenocarcinoma cases were enrolled in the present study. The tumor-node-metastasis (TNM) stages were assigned according to the American Joint Committee on Cancer criteria. All available survival data of the 90 cases were used in the survival analysis. The incomplete tissue points and several missing tissue points were excluded when performing the heat-induced antigen retrieval. Therefore, a total of 68 cases of squamous cell carcinoma and a total of 85 cases of adenocarcinoma were finally included in the statistical analysis. Both Tables 1 and 2 present the detailed clinical parameters of the patients.

**Table 1:** Correlation between TIM-1 expression in lung adenocarcinoma tissues and patients’ clinical parameters

Clinical parameters	Cases	TIM-1 expression level		*χ* ^2^	*P*-value
		Low (H-score ≤ 230)	High (H-score >230)		
Gender				0.063	0.8024
Male	45	35	10		
Female	40	32	8		
Age (years)				0.389	0.5328
< 60	37	28	9		
≥ 60	48	39	9		
Tumor size (cm)				0.329	0.5663
≤ 5	70	56	14		
> 5	15	11	4		
Tumor stage				0.076	0.7832
T_1_ + T_2_	64	50	14		
T_3_ + T_4_	21	17	4		
Lymph node metastasis				1.727	0.1888
No	40	34	6		
Yes	45	33	12		
TNM stage				2.486	0.1148
I + II	47	40	7		
III + IV	38	27	11

**Table 2:** Correlation between TIM-1 expression in lung squamous cell carcinoma tissues and patients’ clinical parameters

Clinical parameters	Cases	TIM-1 expression level		*χ* ^2^	*P*-value
		Low (H-score ≤ 220)	High (H-score > 220)		
Gender				0.021	0.8837
Male	64	50	14		
Female	4	3	1		
Age (years)				0.143	0.7058
< 60	20	15	5		
≥ 60	48	38	10		
Tumor size (cm)				1.418	0.2337
≤ 5	45	37	8		
> 5	23	16	7		
Tumor stage				0.000	0.9844
T_1_ + T_2_	50	39	11		
T_3_ + T_4_	18	14	4		
Lymph node metastasis				1.493	0.2218
No	41	34	7		
Yes	27	19	8		
TNM stage				3.969	*0.0463*
I + II	38	33	5		
III + IV	30	20	10		

Italic signifies *P *< 0.05

### Reagents and cell lines

Rabbit polyclonal antibody against human TIM-1 (PA5-20244) was purchased from Thermo Scientific (Waltham, MA, USA). Rabbit monoclonal antibodies against human PTEN (#9188), human phos-AKT (#9614) and human AKT (#4685) were obtained from Cell Signaling Technology (Danvers, MA, USA). Horseradish peroxidase (HRP)-conjugated goat anti mouse/rabbit secondary antibody (K500711) was supplied by Dako (Glostrup, Denmark). In addition, rabbit antibody against human GAPDH (Sigma, St. Louis, MO, USA) was used as a loading control in Western blotting analysis. The RNeasy Mini Kit was provided by Qiagen (Valencia, CA, USA), and SYBR Green Master Mix kit was purchased from Takara (Dalian, China). RPMI-1640 medium, DMEM medium and fetal bovine serum (FBS) were obtained from Gibco (Cambrex, MD, USA). Human NSCLC cell lines, A549 and SK-MES-1, were supplied by Chinese Academy of Sciences, Shanghai Institutes for Biological Sciences (Shanghai, China). The cell lines were maintained in RPMI-1640 medium or DMEM supplemented with 10% FBS, 100 U/mL benzylpenicillin, 100 μg/mL streptomycin and 2 mM l-glutamine at 37 °C in a humidified environment containing 5% CO_2_.

### Immunohistochemistry (IHC) assay and evaluation of staining intensity

IHC assay was performed according to the methods in our previously published reports [22–24]. The antigen retrieval was conducted by heating the tissue sections at 100 °C for 30 min in EDTA solution (1 mM, pH 9.0). The tissue sections were incubated with primary antibody against human TIM-1 (1:400) at 4 °C overnight, followed by incubation of HRP-conjugated goat anti mouse/rabbit secondary antibody. All slides were blindly examined by two independent senior pathologists. The immunostaining intensity of TIM-1 was assessed according to the *H*-*score* method as previously described [22, 24]: *H*-*score* = (% unstained tumor cells × 0) + (% weakly stained tumor cells x1) + (% moderately stained tumor cells × 2) + (% strongly stained tumor cells x3). The *H*-*scores* ranged from 0 (100% negative tumor cells) to 300 (100% strongly stained tumor cells). The scoring results from the two pathologists were averaged and used for statistical analysis.

### RNAi lentivirus generation and infection

Small hairpin RNA (shRNA) targeting human TIM-1 gene (NM_012206.2; GenBank) was obtained from Shanghai Generay Biotech Co., Ltd. (Shanghai, China) and cloned into a lentiviral vector pLV-U6-GFP. The shRNA target sequence against TIM-1 was as follows: 5′-ACGACTGTTCTGACGACAATG-3′. The recombinant TIM-1-targeting lentivirus (LV-TIM-1-shRNA virus) and control mock lentivirus (LV-NC virus) were prepared and transfected into A549 or SK-MES-1 cells. The infected cells were analyzed by flow cytometry (Canto II, BD, USA), and the GFP-positive cells from the two groups were subsequently sorted using an Aria II flow sorter (BD Bioscience, NJ, USA).

### Real-time polymerase chain reaction (RT-PCR)

RT-PCR was used to examine the expression of TIM-1 at the mRNA level in A549 or SK-MES-1 cell between LV-TIM-1-shRNA and LV-NC groups. Briefly, total RNA was extracted from various cell lines by TRIzol reagent (Invitrogen, USA), and PCR was performed on an ABI 7600 System (Applied Biosystems, USA) according to the manufacturer’s instructions. The primer sequences for housekeeping gene (GAPDH) and target gene (TIM-1) were listed as follows: GAPDH forward primer: 5′-TGACTTCAACAGCGACACCCA-3′, GAPDH reverse primer: 5′-CACCCTGTTGCTGTAGCCAAA-3′; TIM-1 forward primer: 5′-TACCCTGTATCAGGACCAGGA-3′, TIM-1 reverse primer: 5′-GAGAGCTCTGTGCCTTCCAA-3′. The relative mRNA expression level of TIM-1 was calculated using the 2^−ΔΔCT^ method.

### Western blotting analysis

Western blotting analysis was used to detect the expressions of TIM-1, PTEN, phos-AKT and total AKT at the protein level in different cellular models as previously described [22, 24].

### Cellular studies of cell viability, migration, invasion and cell cycle

The effects of TIM-1 depletion on biological functions of NSCLC cell lines were assessed according to our published reports [22, 24]. Briefly, the cell viability was examined using Cell Counting Kit-8 (CCK-8, Beyotime, Shanghai, China). The cell migration ability was evaluated by wound-healing assay, the cell invasion ability was investigated by transwell assay, and the cell cycle was assessed by the flow cytometry following propidium iodide staining.

### Agilent microarray analysis

Purified RNA was labeled and hybridized onto the Agilent Human Gene Expression Analysis platform (8*60 K, Design ID: 039494) provided by Oebiotech Co., Ltd. (Shanghai, China). Differentially expressed genes (DEGs) were then identified based on a threshold setting of fold change ≥ 2.0. Afterwards, Gene ontology (GO) and Kyoto Encyclopedia of Genes and Genomes (KEGG) analyses were applied to determine the roles of these DEGs.

### Statistical analysis

Data were expressed as the mean and range or mean ± SD of three independent experiments. Statistical analysis was conducted using the paired Student’s *t*-test, the Wilcoxon signed-rank test, the Chi-square test or the Log-rank survival analysis where appropriate for final analysis of the data. All the statistical analyses were performed using the GraphPad Prism 5.0 software package (GraphPad Software, Inc., San Diego, USA). A *P *< 0.05 was considered as statistically significant.

## Results

### TIM-1 expression in NSCLC tissues and adjacent normal tissues

**Fig. 1:**
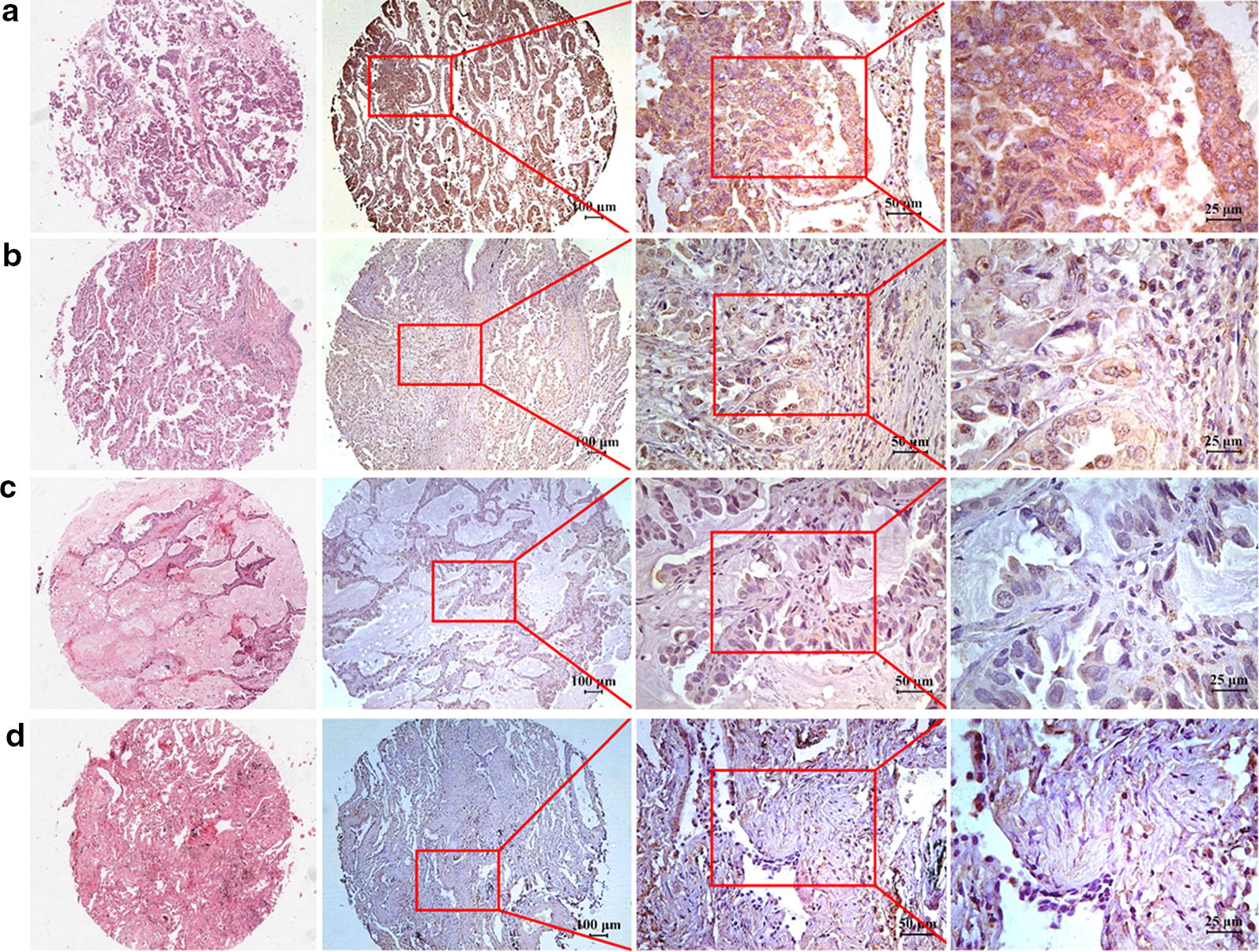
TIM-1 expression in human lung adenocarcinoma tissues and adjacent normal tissues. **a**–**c** Positive TIM-1 immunostaining could be found in the cytoplasm and on the membrane of cancer cells in lung adenocarcinoma tissues (**a** strong, **b** moderate, **c** weak). **d** Weak or negative staining of TIM-1 immunostaining could be found in normal tissues in adjacent normal tissues

**Fig. 2:**
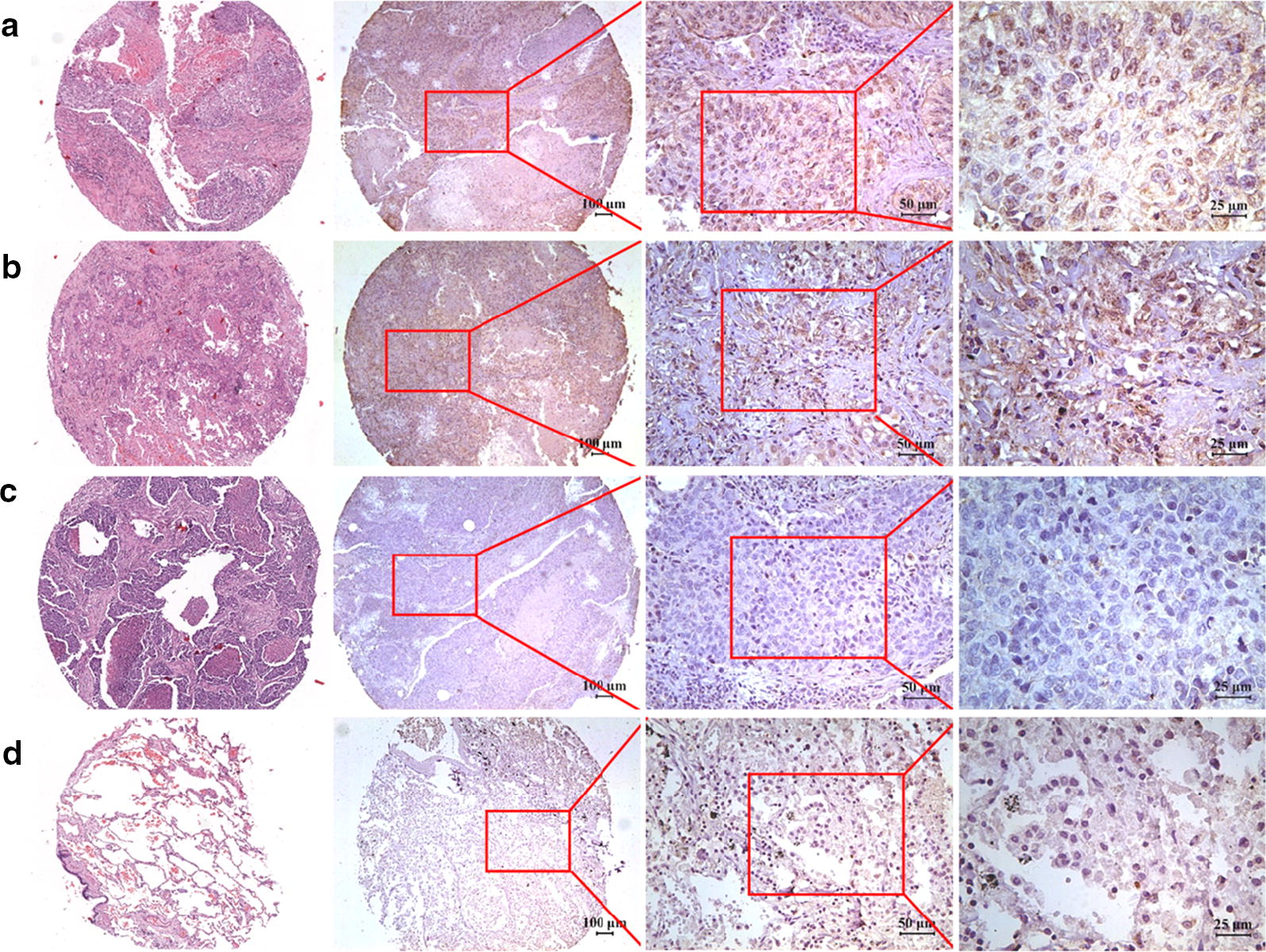
TIM-1 expression in human lung squamous cell carcinoma tissues and adjacent normal tissues. **a**–**c** Positive TIM-1 immunostaining could be found in the cytoplasm and on the membrane of cancer cells in lung squamous cell carcinoma tissues (**a** strong, **b** moderate, **c** weak). **d** Weak or negative staining of TIM-1 immunostaining could be found in normal tissues in adjacent normal tissues

**Fig. 3:**
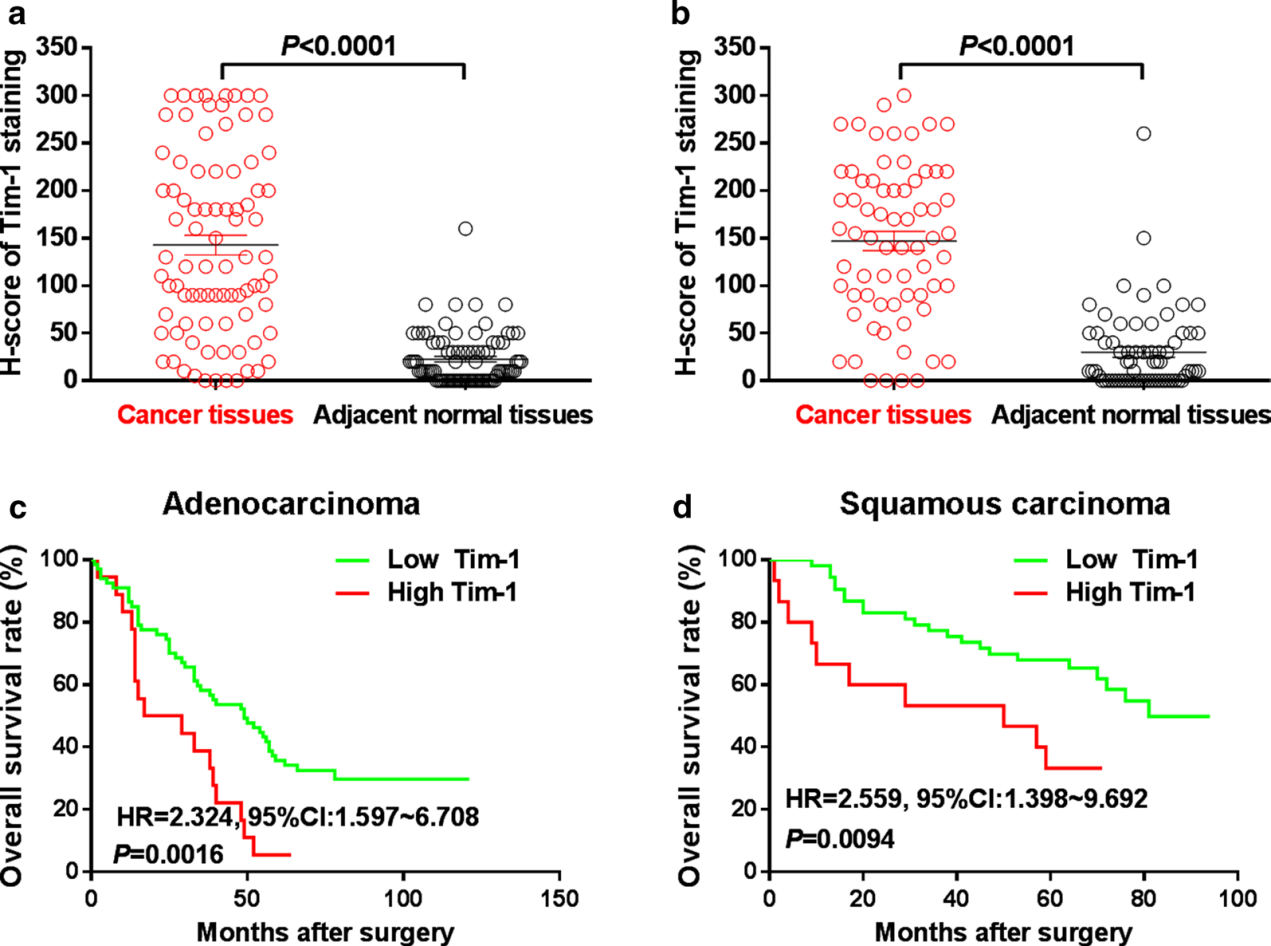
Prognostic value of TIM-1 expression in NSCLC. **a** The median *H*-*score* of TIM-1 expression in lung adenocarcinoma tissues was 220 (0–300), while it was 10 (0–160) in adjacent normal tissues. **b** The median *H*-*score* of TIM-1 expression in lung squamous cell carcinoma tissues was 152.5 (0–300), while it was 10 (0–260) in adjacent normal tissues. **c** The OS rate of lung adenocarcinoma patients with higher TIM-1 expression level (*H*-*score *> 230) was significantly lower compared with the patients with lower TIM-1 expression level (*H*-*score *≤ 230) (*P *= 0.0016, HR = 2.324, 95% CI 1.597–6.708). **d** The OS rate of lung squamous cell carcinoma patients with higher TIM-1 expression level (*H*-*score *> 220) was significantly lower compared with the patients with lower TIM-1 expression level (*H*-*score *≤ 220) (*P *= 0.0094, HR = 2.559, 95% CI 1.398–9.692)

The IHC assay was performed to examine the expression pattern of TIM-1 at the protein level in human NSCLC tissues and adjacent normal tissues. Figure 1a–c illustrate that positive TIM-1 immunostaining could be found in the cytoplasm and on the membrane of cancer cells in lung adenocarcinoma tissues, while weak or negative staining of TIM-1 was detected in adjacent normal tissues (Fig. 1d). Figure 2a–c shows that positive TIM-1 immunostaining was observed in the cytoplasm and on the membrane of cancer cells in lung squamous cell carcinoma tissues, while weak or negative staining of TIM-1 immunostaining was found in adjacent normal tissues (Fig. 2d). The median *H*-*score* of TIM-1 expression in lung adenocarcinoma tissues was 220 (0–300), while it was 10 (0–160) in adjacent normal tissues (Fig. 3a). The median *H*-*score* of TIM-1 expression in lung squamous cell carcinoma tissues was 152.5 (0–300), while it was 10 (0–260) in adjacent normal tissues (Fig. 3b).

### Prognostic value and clinical implications of TIM-1 expression in human NSCLC

**Fig. 4:**
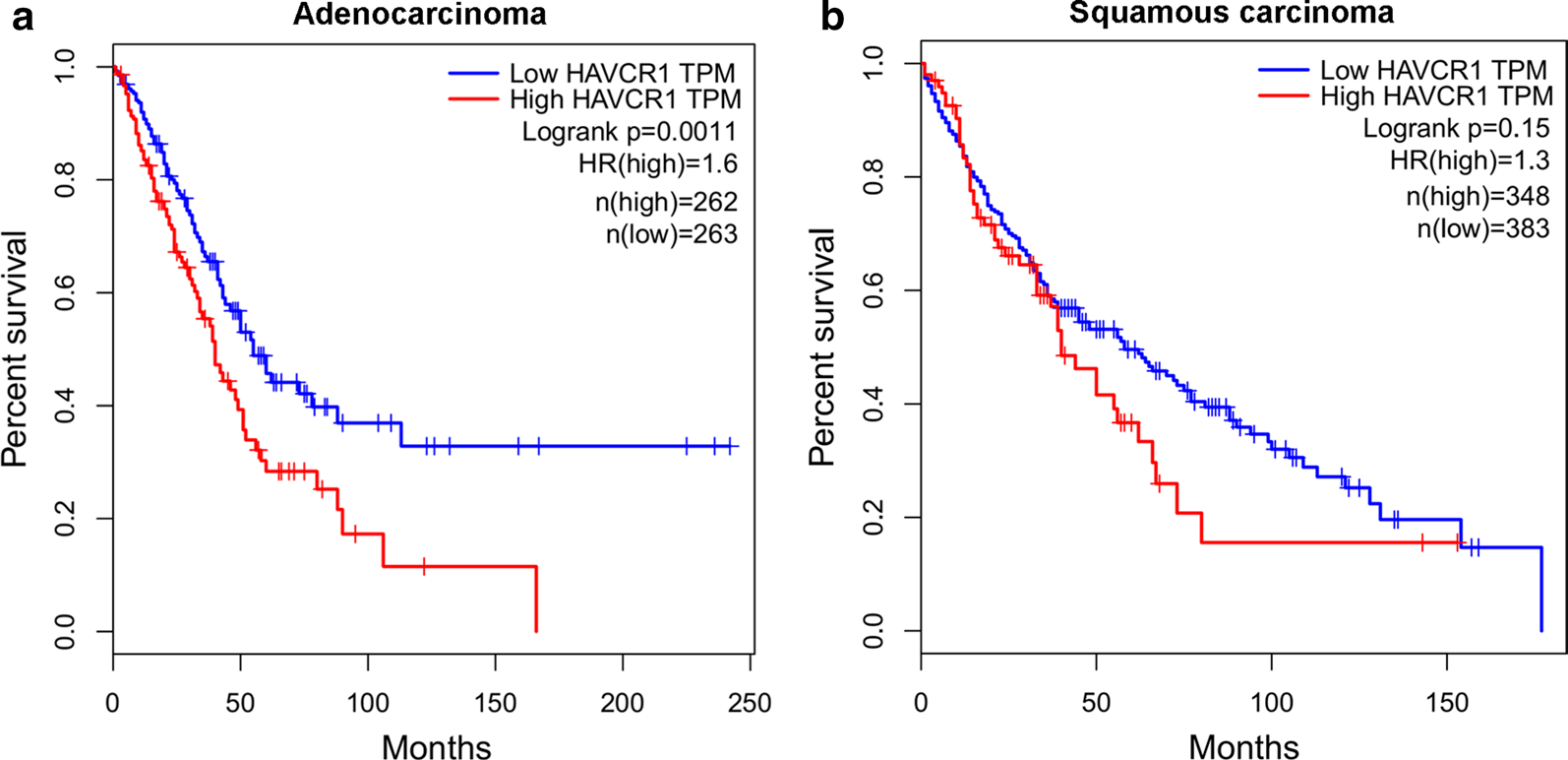
Prognostic value of TIM-1 expression at the mRNA level in NSCLC based on the TCGA data. **a** Based on the TCGA data from http://gepia.cancer-pku.cn/, we also found that the OS rate of lung adenocarcinoma patients with higher TIM-1 mRNA expression level was significantly lower compared with the patients with lower TIM-1 mRNA expression level (*P *= 0.0011). **b** The OS rate of lung squamous cell carcinoma patients with higher TIM-1 mRNA expression level also trends to be lower than that of the patients with lower TIM-1 mRNA expression level (*P *= 0.15)

Figure 3c shows that, the OS rate of lung adenocarcinoma patients with higher TIM-1 expression level (*H*-*score *> 230) was significantly lower compared with the patients with lower TIM-1 expression level (*H*-*score *≤ 230) (*P *= 0.0016, HR = 2.324, 95% CI 1.597–6.708). Figure 3d shows that, the OS rate of lung squamous cell carcinoma patients with higher TIM-1 expression level (*H*-*score *> 220) was significantly poorer compared with the patients with lower TIM-1 expression level (*H*-*score *≤ 220) (*P *= 0.0094, HR = 2.559, 95% CI 1.398–9.692). We could not find any significant associations between TIM-1 expression in lung adenocarcinoma tissues and any clinical parameters (Table 1). Higher TIM-1 expression in lung squamous cell carcinoma tissues is significantly correlated with advanced TNM stage (*χ*^2^ = 3.969, *P *= 0.0463, Table 2), but not any other parameters. COX model also shows that, higher TIM-1 expression in lung adenocarcinoma tissues (Table 3, *P *= 0.012) and in squamous cell carcinoma (Table 4, *P *= 0.050) could be used as independent prognostic predictor for the patients respectively. Moreover, based on the TCGA data from http://gepia.cancer-pku.cn/, we also find that the OS rate of lung adenocarcinoma patients with higher TIM-1 mRNA expression level is significantly poorer compared with the patients with lower TIM-1 mRNA expression level (Fig. 4a, *P *= 0.0011), and the OS rate of lung squamous cell carcinoma patients with higher TIM-1 mRNA expression level also trends to be lower than that of the patients with lower TIM-1 mRNA expression level (Fig. 4b, *P *= 0.15).

**Table 3:** Cox model analysis for the correlation between TIM-1 expression level in lung adenocarcinoma and patients’ clinical parameters

Clinical parameters	Uni-variate		Multi-variate	
	HR (95% CI)	*P*	HR (95% CI)	*P*
Gender (M/F)	1.306 (0.793–2.150)	0.294	1.417 (0.827–2.426)	0.204
Age (years) (>60/≤ 60)	0.965 (0.587–1.587)	0.889	1.203 (0.719–2.013)	0.483
Tumor size (>5 cm/≤ 5 cm)	1.761 (0.936–3.314)	0.079	1.483 (0.757–2.904)	0.251
TNM stage (III + IV/I + II)	2.746 (1.641–4.597)	*0.000*	2.698 (1.571–4.634)	*0.000*
TIM-1 expression (high/low)	2.324 (1.597–6.708)	*0.002*	2.109 (1.180–3.767)	*0.012*

Italic signifies *P *< 0.05

**Table 4:** Cox model analysis for the correlation between TIM-1 expression level in lung squamous cell carcinoma and patients’ clinical parameters

Clinical parameters	Uni-variate		Multi-variate	
	HR (95% CI)	*P*	HR (95% CI)	*P*
Gender (M/F)	0.399 (0.139–1.147)	0.088	0.328 (0.100–1.081)	0.067
Age (years) (>60/≤ 60)	2.395 (0.921–6.229)	0.073	2.681 (1.011–7.109)	*0.048*
Tumor size (>5 cm/≤ 5 cm)	0.845 (0.400–1.786)	0.659	0.642 (0.295–1.396)	0.263
TNM stage (III + IV/I + II)	1.963 (0.970–3.969)	0.061	2.343 (0.995–5.515)	0.051
TIM-1 expression (high/low)	2.559 (1.398–9.692)	*0.009*	2.304 (0.999–5.312)	*0.050*

Italic signifies *P *< 0.05

### Depletion of TIM-1 affects cellular functions of NSCLC cells

**Fig. 5:**
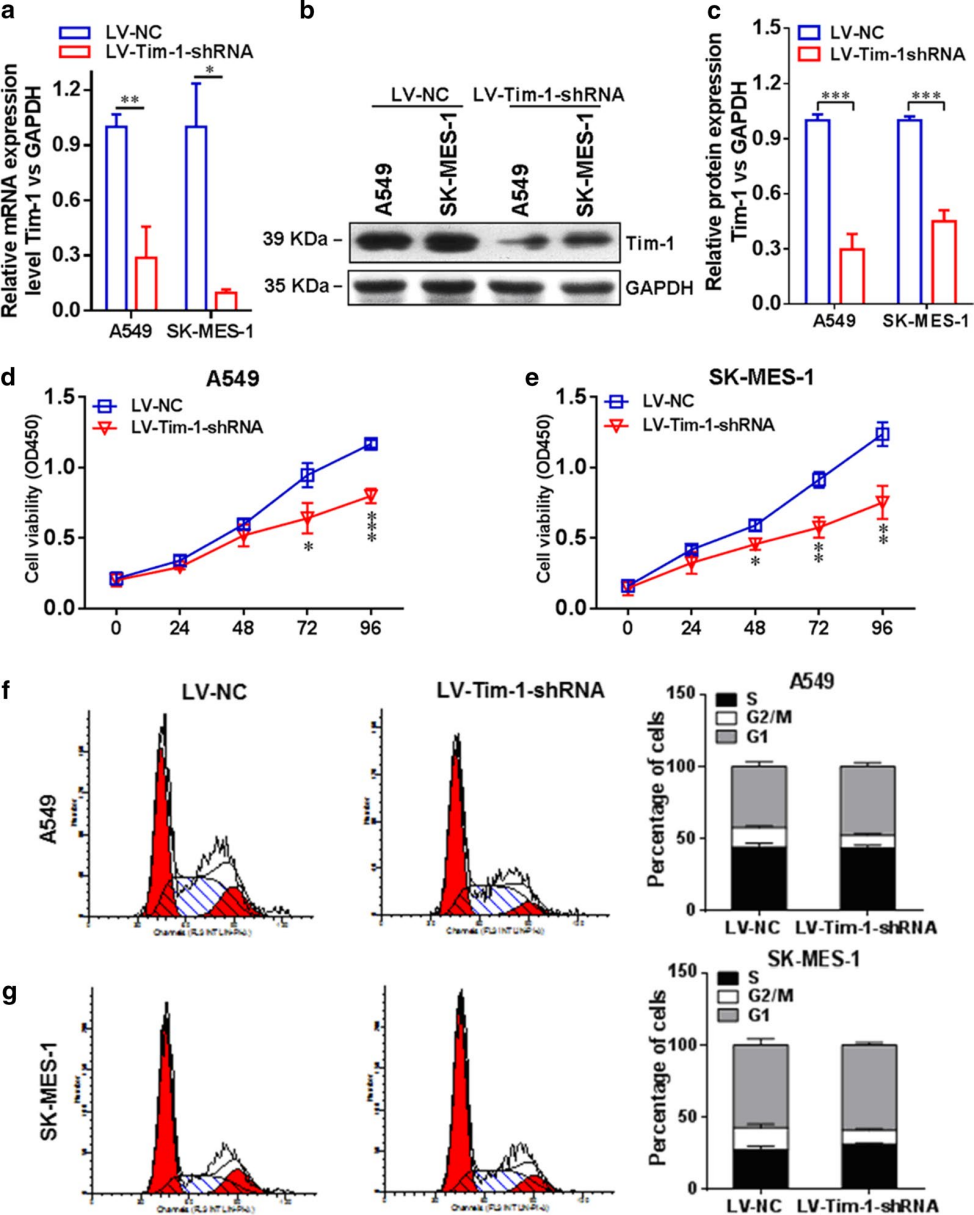
Depletion of TIM-1 in NSCLC cell lines. **a** The expression of TIM-1 at the mRNA level in the LV-TIM-1-shRNA group was significantly lower than that in the LV-NC group, both in A549 (*P *< 0.01) and SK-MES-1 cells (*P *< 0.05). **b**, **c** We also confirmed that the expression of TIM-1 at the protein level in the LV-TIM-1-shRNA group was significantly lower than that in the LV-NC group, both in A549 (*P *< 0.001) and SK-MES-1 cells (*P *< 0.001). **d** In A549 cells, the cell viability of the LV-TIM-1-shRNA group at 72 h (*P *< 0.05) or 96 h (*P *< 0.001) was significantly lower than that of the LV-NC group. **e** In SK-MES-1 cells, the cell viability of the LV-TIM-1-shRNA group at 48 h (*P *< 0.05), 72 h (*P *< 0.01) or 96 h (*P *< 0.01) was significantly lower than that of the LV-NC group. **f**, **g** The cell cycle examination also showed that after depletion of TIM-1, the proportion of G1-phase cells in the LV-TIM-1-shRNA group was significantly increased compared with the LV-NC group, both in A549 and SK-MES-1 cells

**Fig. 6:**
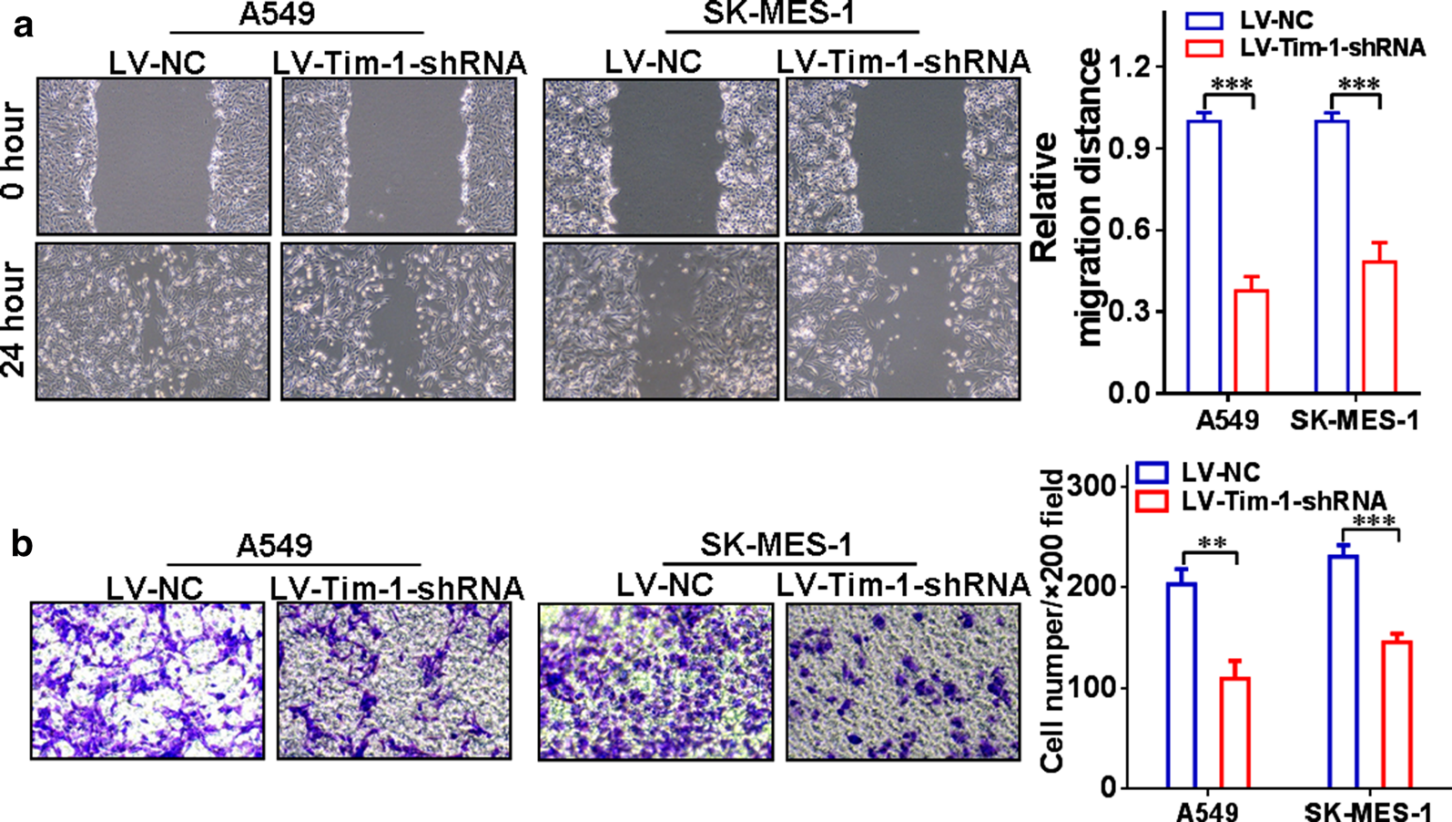
Depletion of TIM-1 affects cellular functions of NSCLC cells. **a** The cell-free area of the LV-TIM-1-shRNA group was significantly narrower than that of the LV-NC group at 24 h (both *P *< 0.001 in A549 and SK-MES-1 cells). **b** Depletion of TIM-1 significantly decreased the number of invaded cells in the LV-TIM-1-shRNA group compared with the LV-NC group (in A549, *P *< 0.01, and in SK-MES-1, *P *< 0.001)

In order to further investigate the essential role of TIM-1 in functional regulation of NSCLC cells, we depleted TIM-1 in both A549 and SK-MES-1 cells by using RNAi methods. Figure 5a indicates that the TIM-1 expression at the mRNA level in the LV-TIM-1-shRNA group was significantly lower than that in the LV-NC group both in A549 (*P *< 0.01) and SK-MES-1 cells (*P *< 0.05). Figure 5b, c display that the TIM-1 expression at the protein level in the LV-TIM-1-shRNA group was significantly lower than that in the LV-NC group both in A549 (*P *< 0.001) and in SK-MES-1 cells (*P *< 0.001). Moreover, the results of CCK-8 assay showed that the cell proliferation rate was significantly decreased upon depletion of TIM-1. Figure 5d reveals that in A549 cells, the cell viability of the LV-TIM-1-shRNA group at 72 h (*P *< 0.05) or 96 h (*P *< 0.001) was significantly lower than that of the LV-NC group at corresponding time points. Figure 5e shows that in SK-MES-1 cells, the cell viability of the LV-TIM-1-shRNA group at 48 h (*P *< 0.05), 72 h (*P *< 0.01) or 96 h (*P *< 0.01) was significantly lower than that of the LV-NC group at corresponding time points. The cell cycle examination also exhibited that the proportion of G1-phase cells in the LV-TIM-1-shRNA group was significantly increased compared with the LV-NC group both in A549 (Fig. 5f) and SK-MES-1 cells (Fig. 5g) upon depletion of TIM-1. In addition, the wound-healing assay and transwell assay were also used to assess the migration and invasion abilities of TIM-1-depleted NSCLC cells, respectively. Figure 6a shows that the cell-free area of the LV-TIM-1-shRNA group was significantly narrower than that of the LV-NC group at 24 h (both *P *< 0.001 in A549 and SK-MES-1 cells). Figure 6b indicates that depletion of TIM-1 significantly decreased the number of invaded cells in the LV-TIM-1-shRNA group compared with the LV-NC group (in A549, *P *< 0.01, and in SK-MES-1, *P *< 0.001).

### PI3K/Akt signaling pathway may be involved in the regulation of TIM-1 in the cellular functions of NSCLC cells

**Fig. 7:**
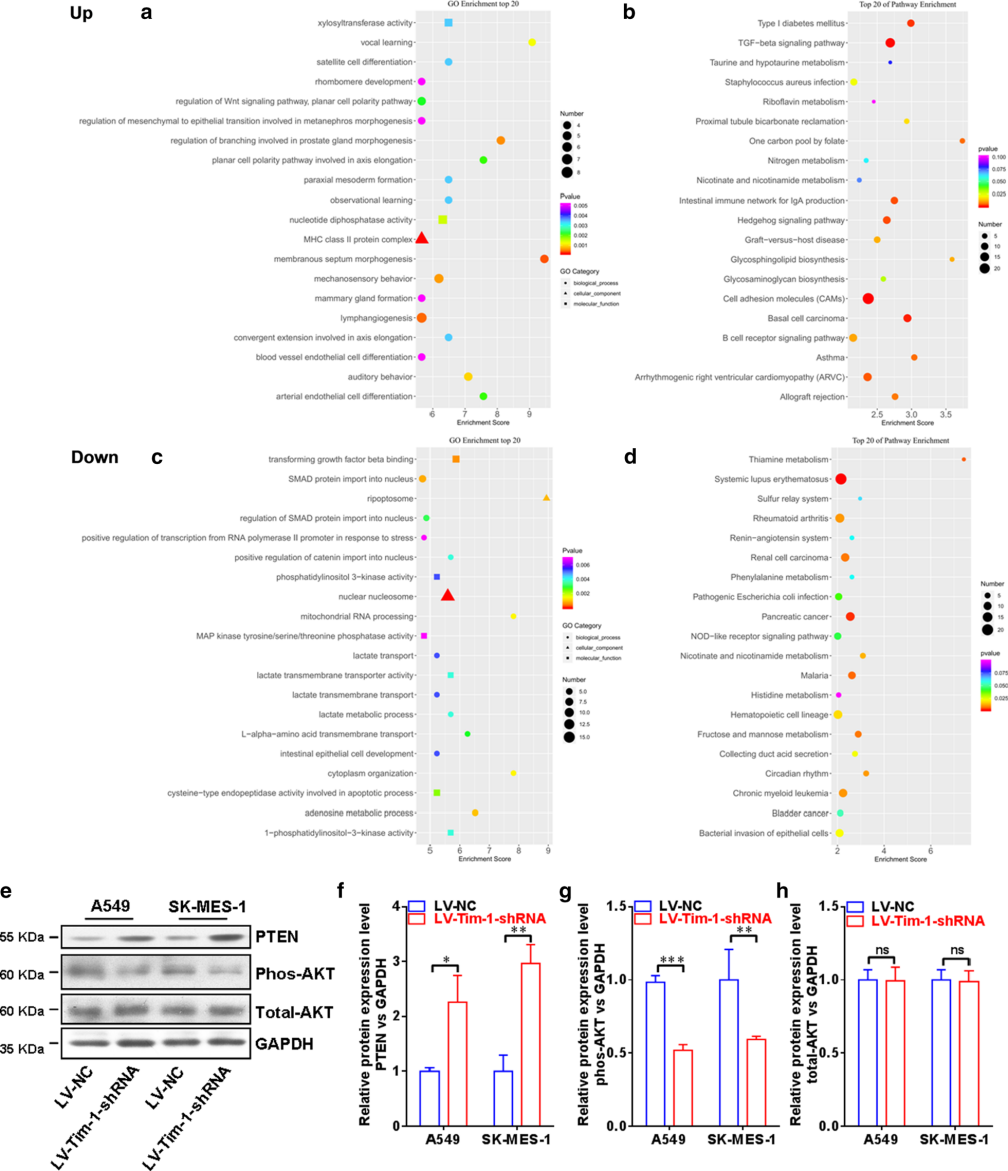
PI3K/Akt signaling pathway may be involved in the regulation of TIM-1 in the cellular functions of NSCLC cells. The Agilent microarray analysis was used to identify the DEGs between the LV-TIM-1-shRNA group and LV-NC group in A549 and SK-MES-1 cells. **a**, **b** The co-up-regulated gene profile. **c**, **d** The co-down-regulated gene profile. Then the expressions of PTEN, phos-AKT and total AKT at the protein level were examined by using Western blotting analysis (**e**). **f**, **g** Demonstrated that the expression of PTEN at the protein level was increased, and the expression of phos-AKT at the protein level was decreased after depletion of TIM-1 in both two cell lines, while no significant change was found in the expression of total AKT at the protein level (**h**)

In order to further unveil the potential mechanism of the regulatory role of TIM-1 in the cellular functions of NSCLC cells, we carried out the Agilent microrray analysis to identify the DEGs between LV-TIM-1-shRNA group and LV-NC group cells in A549 or in SK-MES-1 respectively. As shown in Fig. 7, by using GO or KEGG enrichment methods, both A and B demonstrate the co-up-regulated gene profile, and both C and D demonstrate the co-down-regulated gene profile. Herein, as listed in the top 20 pathways, we selected PI3K/Akt pathway to further verify that whether this signaling pathway was involved in the TIM-1-mediated regulation in NSCLC cells. Then the protein levels of the related molecules such as PTEN, phos-AKT and total-AKT were examined by using western blotting (Fig. 7e). Figure 7f, g demonstrate that, the protein level of PTEN is increased, and the protein level of phos-AKT is decreased after depletion of TIM-1 expression in both two cells lines, while no significant change is found at the protein level of total-AKT (Fig. 7h).

## Discussion

TIM-1, a member from TIM family, has been well-known as an important co-stimulatory molecule, which is found to be expressed on the cell surface of T cells and dendritic cells [25]. TIM family members are usually up-regulated in activated Th1 or Th2 cells, and they can be used as important surface markers for Th1/Th2 cells [26]. As of now, TIM-4 or phosphatidylserine (PS) has been characterized as the ligands for TIM-1, indicating the functional diversity of this molecule via different pathways [18, 27]. Of note, some data also demonstrate that TIM-1 may have a novel function as part of the regulatory apparatus for tight junction of endothelial cells [28]. Moreover, increased TIM-1 expression is also found in certain human cancer tissues, such as gastric cancer, clear renal cell carcinoma, LCS, primary central nervous system lymphoma, colorectal cancer and so on, and the expression level of TIM-1 in cancer tissues or cancer cells is significantly correlated with cancer progression and survival of the patients [19–21, 29, 30]. Interestingly, some data have also reported that activation of TIM-1 signaling by using TIM-4 fusion protein can lead to the cell apoptosis in colon cancer cells, suggesting a different role of TIM-1 in defining cell fate of cancer cells [31].

In our present study, we firstly found that the TIM-1 expression was increased in NSCLC tissues compared with the adjacent normal tissues, and the OS rate of lung cancer patients (both adenocarcinoma and squamous cell carcinoma) with higher TIM-1 expression was significantly lower compared with the patients with lower TIM-1 expression. The COX model also showed that higher TIM-1 expression in lung cancer tissues could be used as an independent prognostic predictor for the patients suffering from lung adenocarcinoma or lung squamous cell carcinoma. Furthermore, we depleted TIM-1 in lung cancer cell lines A549 and SK-MES-1. Our cellular functional studies also revealed that decreased expression of TIM-1 could significantly inhibit the cell viability as well as the abilities of migration and invasion. In addition, our microarray data revealed that certain signal pathways were altered and enriched after depletion of TIM-1. We subsequently verified that PI3K/Akt pathway was involved in the TIM-1-mediated regulation of cellular functions in NSCLC cells. It has been demonstrated that in T cell cells, the stimulation of TIM-1 can recruit p85 adaptor subunits of PI3K and then promote the T cell activation via PI3K pathway [32].

Recently, the strategy by targeting TIM-1 on tumor cells holds a great promise for therapeutic treatment of malignancies. Thomas et al. [33] have reported that antibody–drug conjugate (anti-TIM-1 antibody covalently linked to monomethyl auristatin E) has significant anti-tumor effect on TIM-1-expressing tumors both in vitro and in vivo, including lung cancer. Therefore, based on our present clinical study and cellular investigation, our findings further supported the notion that TIM-1 could serve as a potential therapeutic target for NSCLC.

## Conclusions

Our findings demonstrated that abnormal TIM-1 expression was involved in the progression of human NSCLC, and supported the notion that TIM-1 could serve as an important prognostic risk factor for NSCLC patients.

## Data Availability

The datasets supporting the conclusions of this article are included within the article.

## Abbreviations

TIM-1: T-cell immunoglobulin and mucin domain 1
NSCLC: non-small-cell lung carcinoma
OS: overall survival
IHC: immunohistochemistry
RT-PCR: real-time polymerase chain reaction
CCK-8: Cell Counting Kit-8
DEGs: differentially expressed genes
PS: phosphatidylserine

## References

[1] Siegel RL, Miller KD, Jemal A (2018). Cancer statistics, 2018. CA Cancer J Clin.

[2] Bray F, Ferlay J, Soerjomataram I, Siegel RL, Torre LA, Jemal A (2018). Global cancer statistics 2018: GLOBOCAN estimates of incidence and mortality worldwide for 36 cancers in 185 countries. CA Cancer J Clin.

[3] Chen W, Zheng R, Baade PD, Zhang S, Zeng H, Bray F, Jemal A, Yu XQ, He J (2016). Cancer statistics in China, 2015. CA Cancer J Clin.

[4] Zhang G, Xu Y, Lu X, Huang H, Zhou Y, Lu B, Zhang X (2009). Diagnosis value of serum B7-H3 expression in non-small cell lung cancer. Lung Cancer (Amsterdam, Netherlands).

[5] Vansteenkiste J, Crino L, Dooms C, Douillard JY, Faivre-Finn C, Lim E, Rocco G, Senan S, Van Schil P, Veronesi G (2014). 2nd ESMO consensus conference on lung cancer: early-stage non-small-cell lung cancer consensus on diagnosis, treatment and follow-up. Ann Oncol.

[6] Hirsch FR, Scagliotti GV, Mulshine JL, Kwon R, Curran WJ, Wu YL, Paz-Ares L (2017). Lung cancer: current therapies and new targeted treatments. Lancet.

[7] Bradley JD, Paulus R, Komaki R, Masters G, Blumenschein G, Schild S, Bogart J, Hu C, Forster K, Magliocco A (2015). Standard-dose versus high-dose conformal radiotherapy with concurrent and consolidation carboplatin plus paclitaxel with or without cetuximab for patients with stage IIIA or IIIB non-small-cell lung cancer (RTOG 0617): a randomised, two-by-two factorial phase 3 study. Lancet Oncol.

[8] Senan S, Brade A, Wang LH, Vansteenkiste J, Dakhil S, Biesma B, Martinez Aguillo M, Aerts J, Govindan R, Rubio-Viqueira B (2016). PROCLAIM: randomized phase III trial of pemetrexed-cisplatin or etoposide-cisplatin plus thoracic radiation therapy followed by consolidation chemotherapy in locally advanced nonsquamous non-small-cell lung cancer. J Clin Oncol.

[9] Topalian SL, Hodi FS, Brahmer JR, Gettinger SN, Smith DC, McDermott DF, Powderly JD, Carvajal RD, Sosman JA, Atkins MB (2012). Safety, activity, and immune correlates of anti-PD-1 antibody in cancer. N Engl J Med.

[10] Brahmer JR, Tykodi SS, Chow LQ, Hwu WJ, Topalian SL, Hwu P, Drake CG, Camacho LH, Kauh J, Odunsi K (2012). Safety and activity of anti-PD-L1 antibody in patients with advanced cancer. N Engl J Med.

[11] Hellmann MD, Ciuleanu TE, Pluzanski A, Lee JS, Otterson GA, Audigier-Valette C, Minenza E, Linardou H, Burgers S, Salman P (2018). Nivolumab plus ipilimumab in lung cancer with a high tumor mutational burden. N Engl J Med.

[12] Ngiow SF, von Scheidt B, Akiba H, Yagita H, Teng MW, Smyth MJ (2011). Anti-TIM3 antibody promotes T cell IFN-gamma-mediated antitumor immunity and suppresses established tumors. Can Res.

[13] Nirschl CJ, Drake CG (2013). Molecular pathways: coexpression of immune checkpoint molecules: signaling pathways and implications for cancer immunotherapy. Clin Cancer Res.

[14] Rennert PD (2011). Novel roles for TIM-1 in immunity and infection. Immunol Lett.

[15] Du P, Xiong R, Li X, Jiang J (2016). Immune regulation and antitumor effect of TIM-1. J Immunol Res.

[16] Kaplan G, Totsuka A, Thompson P, Akatsuka T, Moritsugu Y, Feinstone SM (1996). Identification of a surface glycoprotein on African green monkey kidney cells as a receptor for hepatitis A virus. EMBO J.

[17] Feigelstock D, Thompson P, Mattoo P, Zhang Y, Kaplan GG (1998). The human homolog of HAVcr-1 codes for a hepatitis A virus cellular receptor. J Virol.

[18] Kobayashi N, Karisola P, Pena-Cruz V, Dorfman DM, Jinushi M, Umetsu SE, Butte MJ, Nagumo H, Chernova I, Zhu B (2007). TIM-1 and TIM-4 glycoproteins bind phosphatidylserine and mediate uptake of apoptotic cells. Immunity.

[19] Li J, Cao D, Guo G, Wu Y, Chen Y (2013). Expression and anatomical distribution of TIM-containing molecules in Langerhans cell sarcoma. J Mol Histol.

[20] Liu L, Song Z, Zhao Y, Li C, Wei H, Ma J, Du Y (2018). HAVCR20 expression might be a novel prognostic factor for gastric cancer. PLoS ONE.

[21] Mijuskovic M, Stanojevic I, Milovic N, Cerovic S, Petrovic D, Maksic D, Kovacevic B, Andjelic T, Aleksic P, Terzic B (2018). Tissue and urinary KIM-1 relate to tumor characteristics in patients with clear renal cell carcinoma. Int Urol Nephrol.

[22] Chen L, Zhai W, Zheng X, Xie Q, Zhou Q, Tao M, Zhu Y, Wu C, Jiang J (2018). Decreased IFIT2 expression promotes gastric cancer progression and predicts poor prognosis of the patients. Cell Physiol Biochem.

[23] Schalper KA, Carvajal-Hausdorf D, McLaughlin J, Altan M, Velcheti V, Gaule P, Sanmamed MF, Chen L, Herbst RS, Rimm DL (2017). Differential expression and significance of PD-L1, IDO-1, and B7-H4 in human lung cancer. Clin Cancer Res.

[24] Chen L, Sun J, Wu H, Zhou S, Tan Y, Tan M, Shan B, Lu B, Zhang X (2011). B7-H4 expression associates with cancer progression and predicts patient’s survival in human esophageal squamous cell carcinoma. Cancer Immunol Immunother.

[25] McIntire JJ, Umetsu SE, Akbari O, Potter M, Kuchroo VK, Barsh GS, Freeman GJ, Umetsu DT, DeKruyff RH (2001). Identification of Tapr (an airway hyperreactivity regulatory locus) and the linked Tim gene family. Nat Immunol.

[26] Rodriguez-Manzanet R, DeKruyff R, Kuchroo VK, Umetsu DT (2009). The costimulatory role of TIM molecules. Immunol Rev.

[27] Meyers JH, Chakravarti S, Schlesinger D, Illes Z, Waldner H, Umetsu SE, Kenny J, Zheng XX, Umetsu DT, DeKruyff RH (2005). TIM-4 is the ligand for TIM-1, and the TIM-1-TIM-4 interaction regulates T cell proliferation. Nat Immunol.

[28] Martin TA, Harrison GM, Mason MD, Jiang WG (2011). HAVcR-1 reduces the integrity of human endothelial tight junctions. Anticancer Res.

[29] Kishimoto W, Nishikori M, Arima H, Miyoshi H, Sasaki Y, Kitawaki T, Shirakawa K, Kato T, Imaizumi Y, Ishikawa T (2016). Expression of Tim-1 in primary CNS lymphoma. Cancer Med.

[30] Wang Y, Martin TA, Jiang WG (2013). HAVcR-1 expression in human colorectal cancer and its effects on colorectal cancer cells in vitro. Anticancer Res.

[31] Wang H, Zhang X, Sun W, Hu X, Li X, Fu S, Liu C (2016). Activation of TIM1 induces colon cancer cell apoptosis via modulating Fas ligand expression. Biochem Biophys Res Commun.

[32] de Souza AJ, Oak JS, Jordanhazy R, DeKruyff RH, Fruman DA, Kane LP (2008). T cell Ig and mucin domain-1-mediated T cell activation requires recruitment and activation of phosphoinositide 3-kinase. J Immunol.

[33] Thomas LJ, Vitale L, O’Neill T, Dolnick RY, Wallace PK, Minderman H, Gergel LE, Forsberg EM, Boyer JM, Storey JR (2016). Development of a novel antibody-drug conjugate for the potential treatment of ovarian, lung, and renal cell carcinoma expressing TIM-1. Mol Cancer Ther.

